# Notes and News

**Published:** 1904-08-06

**Authors:** 


### Notes and News.

An Infectious Diseases Hospital has been opened at Rattray, in East Perthshire, to serve the burghs of Alyth, Blairgowrie, Coupar-Angus, and Rattray.

The Shand children's ward for 28 cots at the Cardiff Infirmary is now nearly complete, and Miss Thomas, of Hwynmadoc, has contributed £100 towards its equipment.

The foundation-stone of an isolation hospital for the Runcorn Rural District Council has been laid at Dutton. The plans provide for a hospital of 24 beds, distributed in separate blocks, and situated upon a site of eight acres. The initial outlay on the buildings is estimated at £6,455; and the cost of the land was £1,050.

The late Mr. G. Handyside, of Newcastle-on-Tyne, has bequeathed £20,000 to the Newcastle Royal Infirmary, £10,000 each to Newcastle Dispensary, the Whitley Convalescent Home, and the Gateshead Children's Hospital, and £5,000 each to the Newcastle Eye Infirmary and the Newcastle Cathedral Nurses' Loan Society for the Sick Poor.

The Cromer Cottage Hospital, which had been closed for extensions, has been reopened. A new wing has been added to the right of the entrance. This provides two wards, containing 15 beds in all, and an operating room and offices. There have been extensions in the domestic department and other improvements effected. In the male ward a handsome clock has been placed, which has been presented by the patients.

The Board of Management of the East London Hospital for Children state in their half-yearly report to the Governors, that practically no response has been made to their appeal for £500 to start a milk depôt in connection with the hospital. The scheme is, therefore, in abeyance. This is the more to be regretted, since it points to the conclusion that the vital importance to the nation of the proper feeding of infants, notwithstanding all that has been said and written on the subject, is not yet understood or appreciated by the public

Lord Brassey has laid the foundation-stone of a school hall for the Homes for Epileptic Children at the Farm Training Colony at Lingfield.

At the last meeting of the Aberdeen Royal Infirmary a bequest of £1,000 from the late Sir William Henderson was announced. The sum is to be devoted to the endowment of a bed to bear his name.

Important alterations and improvements will be carried out this summer at the National Sanatorium for Consumption at Bournemouth. About 12 more beds will be added and the accommodation for nurses will be improved.

The late Alderman Carter, of Middlesbrough, has bequeathed £1,000 to the North Riding Infirmary. Subject to the payment of legacies, the residue of his estate is to be devoted to the establishment and maintenance of a hospital for Middlesbrough.

The late Rev. Ebenezer Smith, of Shrewsbury, has bequeathed £2,000 each to the Poplar Hospital for Accidents, the Salop Infirmary, and the Eye, Ear, and Throat Hospital for Salop and Wales, and £1,000 each to the Middlesex Hospital, the Lambeth Lying-in Hospital, the Royal London Ophthalmic Hospital, the Great Ormond Street Hospital for Children, and the Putney Hospital for Incurables.

The Queen's Memorial Infectious Diseases Hospital at Melbourne has just been completed on the bank of the River Yarra at Fairfield, a suburb of Melbourne. The hospital stands in about 16 acres of land, and consists of six separate buildings. The whole are composed of red brick with tiled roofs, the floors throughout being of asphalt. Accommodation will be provided for 50 patients initially. The want of a hospital of this description has long been felt at Melbourne.

Not long ago a fresh application was made by the British and Foreign Blind Association to the Postmaster-General, asking for reduced rates of postage for the bulky and weighty literature for the blind. The Postmaster-General replied that rates were regulated by Act of Parliament, and as the proposal presented practical difficulties, he did not think he could take any steps to further it. He pointed out that embossed letters of the blind were conveyed at reduced rates. He, at the same time, gave the Association permission to put forward their contentions and proposals in detail. The proposals and arguments in favour of concessions were then very ably and convincingly set out by the Association, only to receive a typical Government red-tape reply, refusing to do anything further. In his letter to the Postmaster-General, the secretary of the Association drew attention to facilities granted by the Customs Department of most countries to the literature of the blind, and by the postal authorities in Switzerland and America. The correspondence between the Association and the Postmaster-General is published, and will afford interesting reading to the intelligent public. We hope the Association will not allow present failure to damp their efforts, as in the future more enlightened officialdom may surmount the inertia of red-tapeism.

